# SARS-CoV-2 viral budding and entry can be modeled using BSL-2 level virus-like particles

**DOI:** 10.1074/jbc.RA120.016148

**Published:** 2020-11-27

**Authors:** Caroline B. Plescia, Emily A. David, Dhabaleswar Patra, Ranjan Sengupta, Souad Amiar, Yuan Su, Robert V. Stahelin

**Affiliations:** Department of Medicinal Chemistry & Molecular Pharmacology, Purdue Institute of Inflammation, Immunology, and Infectious Disease, Purdue University, West Lafayette, Indiana, USA

**Keywords:** coronavirus, electron microscopy (EM), Golgi, membrane bilayer, SARS-CoV-2, virus-like particle (VLP), viral protein, virology, virus, virus assembly, virus budding, virus entry, ACE2, angiotensin-converting enzyme 2, BCA, bicinchoninic acid assay, DAB, 3,3′-Diaminobenzidine, DMEM, Dulbecco’s modified Eagle’s medium, EEA1, early endosome antigen 1, ERGIC, endoplasmic reticulum–Golgi intermediary complex, FBS, fetal bovine serum, LAMP1, lysosomal associated membrane protein 1, MERS-CoV, Middle East respiratory syndrome coronavirus, ORFs, open reading frames, PFA, paraformaldehyde, SARS-CoV-2, severe acute respiratory syndrome coronavirus 2, SEM, scanning electron microscopy, TEM, transmission electron microscopy, VLPs, virus-like particles, WGA, wheat germ agglutinin

## Abstract

Severe acute respiratory syndrome coronavirus 2 (SARS-CoV-2) was first discovered in December 2019 in Wuhan, China, and expeditiously spread across the globe causing a global pandemic. Research on SARS-CoV-2, as well as the closely related SARS-CoV-1 and MERS coronaviruses, is restricted to BSL-3 facilities. Such BSL-3 classification makes SARS-CoV-2 research inaccessible to the majority of functioning research laboratories in the United States; this becomes problematic when the collective scientific effort needs to be focused on such in the face of a pandemic. However, a minimal system capable of recapitulating different steps of the viral life cycle without using the virus’ genetic material could increase accessibility. In this work, we assessed the four structural proteins from SARS-CoV-2 for their ability to form virus-like particles (VLPs) from human cells to form a competent system for BSL-2 studies of SARS-CoV-2. Herein, we provide methods and resources of producing, purifying, fluorescently and APEX2-labeling of SARS-CoV-2 VLPs for the evaluation of mechanisms of viral budding and entry as well as assessment of drug inhibitors under BSL-2 conditions. These systems should be useful to those looking to circumvent BSL-3 work with SARS-CoV-2 yet study the mechanisms by which SARS-CoV-2 enters and exits human cells.

Severe acute respiratory syndrome SARS-coronavirus 2 (SARS-CoV-2) emerged in December 2019 in Wuhan, China, and has since spread around the globe. As of late October 2020, the virus has been detected in 189 different countries and territories with more than 43 million confirmed cases and more than one million attributed fatalities (https://covid19.who.int). The virulence of coronaviruses has previously been observed in severe acute respiratory syndrome coronavirus (SARS-CoV-1) and Middle East Respiratory Syndrome coronavirus (MERS-CoV) outbreaks in the previous 2 decades; however, there still remains no FDA-approved treatment for *any* coronavirus. In order to develop therapeutics, the ability to study viruses must be accessible. Under current circumstances, the authentic live SARS-CoV-2 virus is restricted to BSL-3 containment facilities; while many of these facilities exist and have refocused their collective efforts on SARS-CoV-2 research, the greater scientific community could be vastly helpful in combating this pandemic if such research was BSL-2 compatible.

For instance, BSL-2 models of other difficult-to-work-with BSL-3 and -4 pathogens such as SARS-CoV-1 (1), MERS (2), Ebola virus (3, 4) Marburg virus (4, 5), and Lassa virus (6) have been implemented in the form of virus-like particles (VLPs). Thus, the development of BSL-2 compatible models and assays to study SARS-CoV-2 assembly, budding, and entry, as well as evaluate potential therapeutics, is imperative. In this work, we aimed to develop morphologically and functionally relevant BSL-2-compatible VLPs to model SARS-CoV-2 budding and entry.

SARS-CoV-2 has a positive-sense single-stranded RNA genome of 29.7 kilobases, which shares 79.6% sequence identity with SARS-CoV-1 (7). Both SARS-CoV-1 and SARS-CoV-2 utilize host cell surface receptor angiotensin-converting enzyme 2 (ACE2) to stimulate cellular uptake of bound viral particles (7). Trafficked through the endocytic system of the cells, SARS-CoV-2 is eventually released into the cytoplasm where it utilizes ten open reading frames (ORFs) to encode numerous nonstructural proteins and four structural proteins (8). As has been described for other coronaviruses, the four structural proteins are in ucleoprotein (N), membrane protein (M), envelope protein (E), and spike protein (S) and are presumed responsible for maintaining the structural integrity of the enveloped SARS-CoV-2 virion (8). The M glycoprotein of coronaviruses drives the assembly and formation of progeny viral particles from the endoplasmic reticulum–Golgi intermediary complex (ERGIC) and is the most abundant viral structural protein in the virion (8). M oligomerizes to create a protein lattice across ERGIC membranes and interacts laterally with S and E, the other two viral membrane proteins, which are integrated into the structural matrix at sites of budding (2, 8). The role of E in assembly and budding is enigmatic, though it has been shown to be crucial for proper assembly of SARS-CoV-1 viral particles and serves as a viroporin altering ion transport (2, 8, 9).

The S protein gives coronavirus particles their pronounced crowned (“corona”) structure (8). While dispensable for viral particle assembly and formation in SARS-CoV-1, the incorporation of S is required for progeny viral particles to successfully infect a host cell (1). The final structural protein, N, is responsible for coordinating the viral RNA genome to the structural matrix, which it does through interactions with the cytosolic C-terminal endodomain of M in an RNA-independent manner (2, 8). These interactions between the four structural proteins facilitate the proper assembly, genomic packaging, and budding of progeny coronavirus particles (8). After budding into the ERGIC lumen, progeny viral particles are released from the infected cell by exocytosis (8). While these processes have yet to be fully examined specifically in the SARS-CoV-2 virus, they are likely to follow a similar scheme based upon homology.

Previously, transient coexpression of the four SARS-CoV-1 structural proteins in mammalian cell culture has been shown to produce self-assembling VLPs, which can be collected, purified, and used to study the molecular biology of the virus (1). Specifically, aspects of the viral life cycle such as assembly (2), budding (2, 10), egress (1, 10), and entry (11) have been studied for SARS-CoV-1. While these VLPs were both morphologically and functionally similar to authentic SARS-CoV-1, they do not contain the viral genome, are noninfectious, and thus can be used in a BSL-2 setting.

Until now, SARS-CoV-2 VLPs have only been used to identify M as the driver of viral particle formation (12) and for vaccine development (13); they have yet to be functionalized to study SARS-CoV-2 entry or inhibitors. Instead, the few identified entry inhibitors of SARS-CoV-2 have been evaluated using classical coronavirus assays such as S-mediated cell–cell fusion (14) or pseudotyped VSV vectors (15). VLPs offer a reliable and realistic model of S-mediated fusion and viral entry events in a BSL-2 setting. Herein, we discuss methods for production, purification, validation, and utilization of SARS-CoV-2 VLPs.

## Results

### SARS-CoV-2 VLP production is driven by M coexpression with additional viral proteins

**Figure 1:**
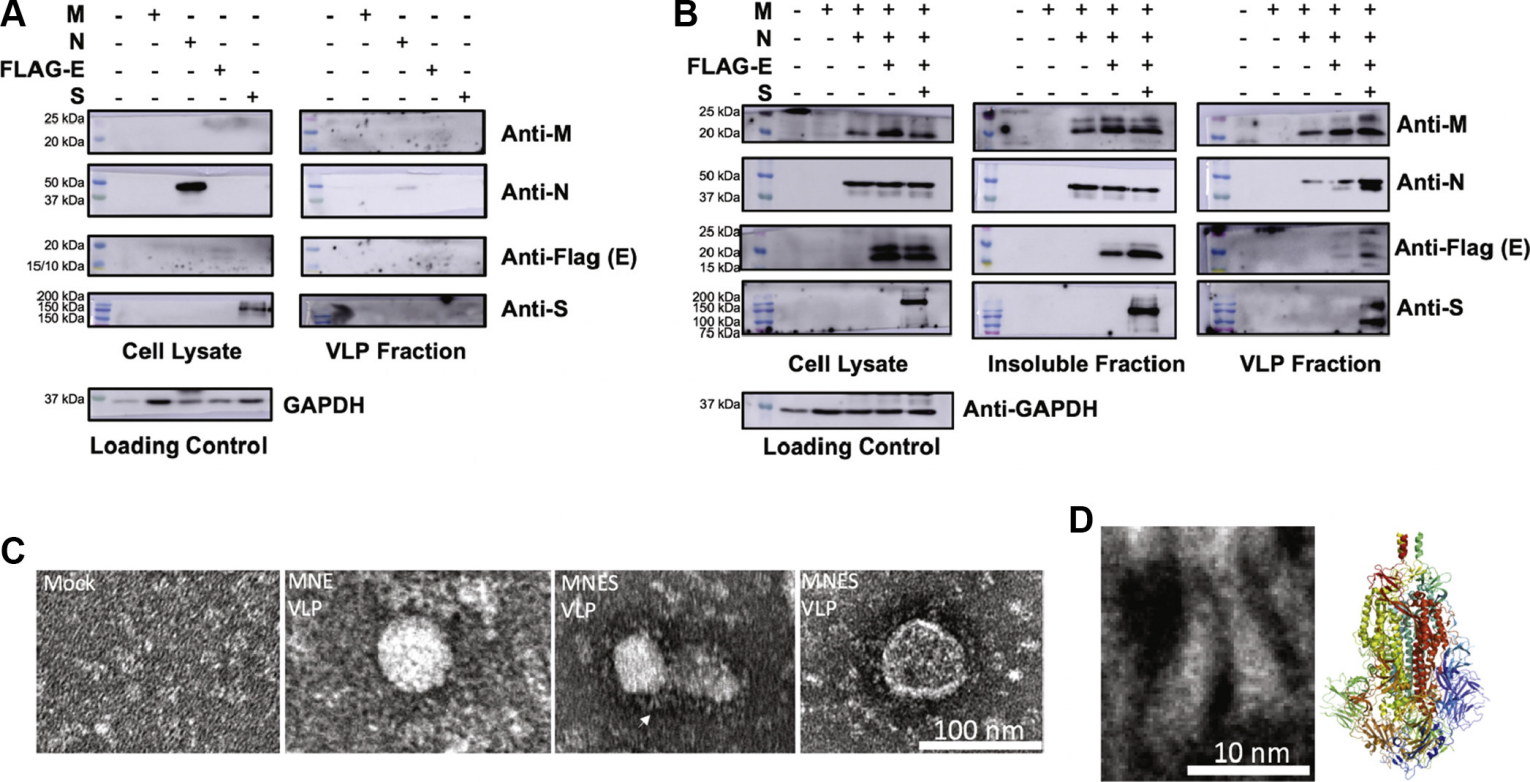
**Production of SARS-CoV-2 virus-like particles (VLPs).***A*, western blot analysis of the cell lysate and VLP fractions of individual expression of the four structural proteins 72-h posttransfection. Total protein content of cell lysates was used to normalize loading conditions and was quantified using the Pierce bicinchoninic acid assay. VLP loading was calculated as a constant ratio to normalized cell lysates. *B*, western blot analysis of the cell lysate and VLP fractions of additive combinations of M, N, and S. Total protein content of the cell lysates was used to normalize loading conditions and was quantified using the Pierce bicinchoninic acid assay. VLP loading was calculated as a constant ratio to normalized cell lysates. *C*, electron microscopy of SARS-CoV-2 VLPs. Purified M + N + E and M + N + E + S VLPs were added to glow discharged 400-mesh copper grids covered with carbon-coated collodion film. Grids were washed in one drop of water, stained in three drops of phosphotungstic acid (1.0% w/v), air dried, and imaged. *White arrow* demarcates zoomed image used for 1D. *D*, two S molecules on the left insert are magnified from marked with *white arrow*. The right insert corresponds to cryo-EM structure of trimeric S protein (PBD: 6ZWV). Its longest dimension is ∼170 Å, which is comparable to negative staining of S protein present around VLPs.

**Figure 2:**
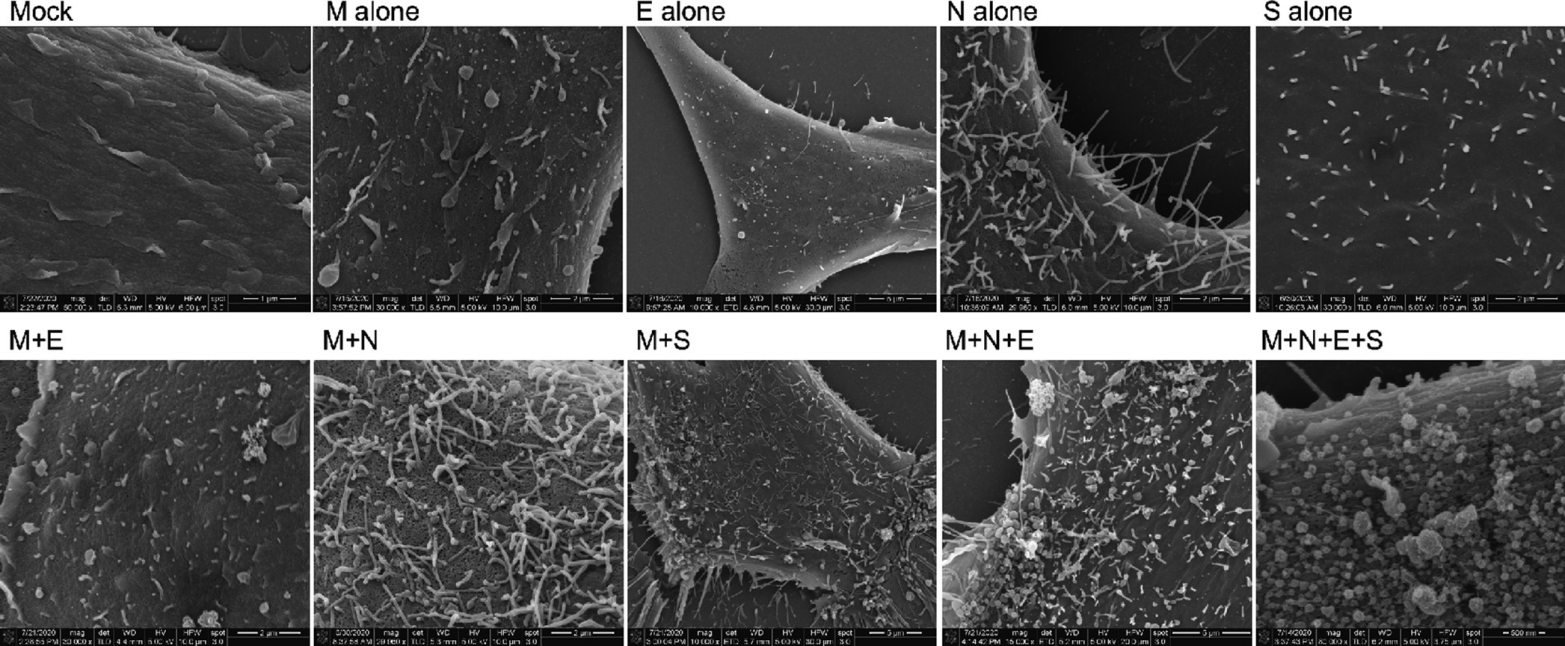
**Scanning electron microscopy of viral structural protein transfected cells.** HEK293 cells were seeded onto coverslips and transfected individually or in combination with M, N, E, and/or S. Cells were fixed with glutaraldehyde 72-h posttransfection and kept at 4 °C until fixed with osmium tetroxide. Samples were then gradually dehydrated with ethanol and completely dehydrated with a critical point dryer. Once dehydrated, samples were mounted onto aluminum pins with double-sided carbon tape, charged with silver paint, and sputter coated prior to imaging. Images range in magnification from 10,000× to 80,000×.

A previous study by Xu *et al.* (12) showed that M was released into the media of HEK293T and Vero E6 cells at 48-h posttransfection independent of other viral structural proteins. We repeated this experiment, independently expressing M, N, E, or S in HEK293 cells and collecting VLPs at 72-h posttransfection. Cell lysates and VLP fractions were analyzed with western blot analysis (Fig. 1*A*). Additionally, we examined independently transfected cells with scanning electron microscopy (SEM) (Fig. 2 and Fig. S1). When analyzed with western blot, M was not detectable in the cell lysate or VLP fraction when expressed independently. The insoluble cellular fraction was evaluated as well, and when expressed alone, M was also undetectable in this fraction (Fig. 1*B*). This is contrary to the findings of Xu *et al*., where Flag-M was detectable in the cell lysate when expressed alone. This discrepancy may be explained by Xu *et al.*’s use of a Flag-tagged construct while we used an untagged construct and the anti-Flag antibody may have superior sensitivity. SARS-CoV-1 M is known to be N-terminally glycosylated and the C-terminal domain is known to be important for interactions with N, which is why we preferred to use the untagged version of M. When coexpressed with N or S, our untagged M was detectable in the cell lysate and VLP fraction (Fig. 1*B* and Fig. S2). This effect of N on M was confirmed with immunofluorescence (Fig. S3). When analyzed with SEM, M independently expressed did appear to induce small changes to membrane structure but overall was qualitatively insignificant compared with mock transfections (Fig. 2).

When independently expressed, N was readily detectable in the cell lysate and a small band was detectable in the VLP fraction (Fig. 1*A*). Intriguingly, N was also found in the insoluble fraction (Fig. 1*B*). When this VLP sample was analyzed with transmission electron microscopy (TEM), this fraction was not found to contain VLPs, but had proteinaceous aggregates that resemble high-order N oligomers (Fig. S4, white arrow). It is possible that what is detected in the western blot was either secreted N protein or detached N-packed filopodia, which cosedimented with VLPs, as SEM analysis revealed that expression of N alone induces filopodia formation. Further, filopodia formation in N-transfected cells was observed with confocal microscopy (Fig. S5). These results are complimentary to a study that previously showed that filaments formed in SARS-CoV-2 infected cells are filled with N protein (16).

When independently expressed, E was not detectable in the cell lysate (Fig. 1*A*). Similar to M, E is enriched by N as it becomes detectable in the cell lysate when coexpressed (Fig. 1*B*, Supporting Information 1). Contradictory to Xu *et al*. and previous work studying other coronavirus VLPs (12, 17, 18), when E was independently expressed, it was not detectable in the VLP fraction by western blot (Fig. 1*A*). Further, TEM analysis of the VLP fraction revealed a proteinaceous background comparable with that of the mock transfected VLP collection. (Fig. S4). SEM analysis of E-transfected cells revealed little to no change in the plasma membrane structure when compared with mock transfected cells (Fig. 2).

When independently expressed, S was readily detectable in the cell lysate but not detectable in the VLP fraction (Fig. 1*A*). Interestingly, SEM analysis of S-transfected cells revealed stiff, rigid protrusions from the plasma membrane (Fig. 2). S trafficking to the plasma membrane was confirmed by confocal microscopy using S-GFP (Fig. S6).

Since none of the viral structural proteins alone could sufficiently support VLP formation, we cotransfected combinations of structural proteins with M, as M is thought to be the major driver of particle assembly in coronaviruses (1, 8, 12). First, combinations of M + N (Fig. 1*B*), M + E (Fig. S2), and M + S (Fig. S2) were cotransfected and VLPs collected 72-h posttransfection. While M remained undetectable in the cell lysate and VLP fraction when expressed alone, when coexpressed with N or S, M was detectable in the cell lysate and VLP fraction. Notably, M + E did not release a detectable level of M. All three of these combinations were also examined with SEM (Fig. S1). M + E did not produce major changes in plasma membrane structure; however, M + N and M + S did. In M + N transfected cells, long filamentous filipodia were observed while in M + S transfected cells, stiff, rigid filaments were observed.

We further explored the triple combination M + N + E (Fig. 1*B*), which produced a detectable level of VLPs. The addition of E to the M + N system enriched VLP production, indicating an important role of E in assembly and release. SEM analysis of M + N + E transfected cells revealed visible changes in membrane structure and VLPs at the cell surface. TEM analysis of the M + N + E VLP fraction revealed bald, spherical VLPs, which were not observed in the mock sample (Fig. 1*C*). When all four structural proteins were expressed and analyzed with TEM, spherical particles of approximately 100 nm in size were observed with a pronounced crown or “corona” (Fig. 1*C*). M + N + E + S transfected cells were also analyzed by SEM and revealed numerous VLPs at the cell surface, specifically at the base of filopodia. This is in agreement with SEM data examining release of SARS-CoV-1 viral particles (19) and recent SEM on SARS-CoV-2 infected cells (16). When analyzed with western blot, the M + N + E + S condition released the most VLPs (Fig. 1*B*). These findings are again contradictory to those of Xu *et al*. where S seemed to limit M release; however, in their study, all four structural proteins harbored a tag in contrast to our system. In the VLP fraction, S appeared as two bands; the second band represents a population of S cleaved by host furin proteases during egress.

### Transient production of VLPs can be used to model viral assembly and budding

To model VLP assembly and budding, we utilized a recent electron microscopy technology, ascorbate peroxidase (APEX2) tagging, by exploiting S protein’s tolerance of a C-terminal tag to produce S-APEX2. Unlike other exogenous peroxidases, APEX2 remains active in the cytosol after TEM processing. APEX2 works by catalyzing 3,3′-Diaminobenzidine (DAB) oxidation, which produces a dark brown precipitate visible with TEM (20).

**Figure 3:**
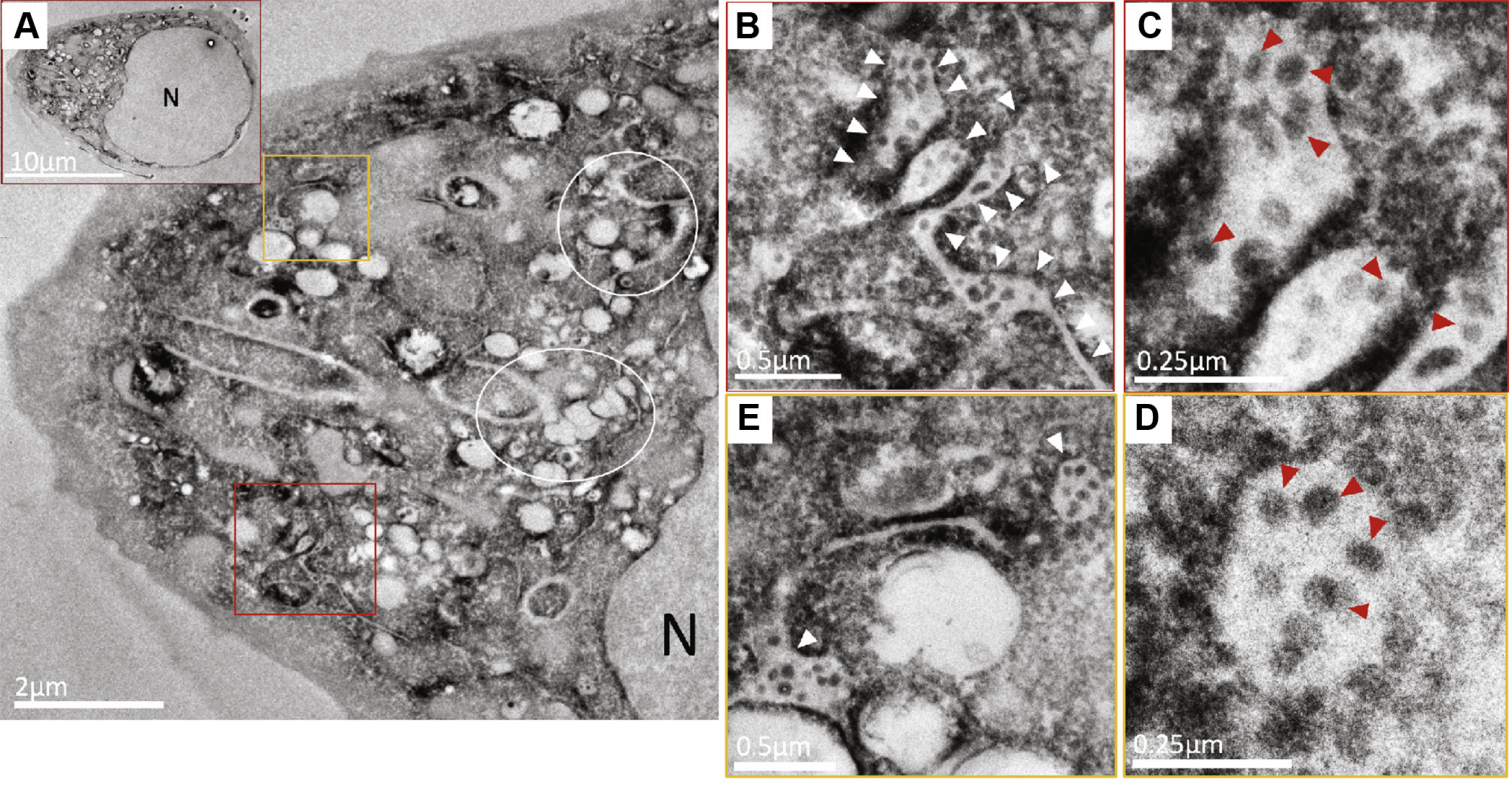
**Detection and visualization of VLP assembly and budding via electron microscopy.** M, N, E, and S-APEX2 were coexpressed in HEK293 cells. Thirty hours posttransfection, cells were fixed and processed for imaging. TEM images of ultrathin sections of resin-embedded cells subjected to APEX2-DAB assay exhibit intense staining of the perinuclear endomembrane system (*A*, *inset*). This region exhibited localized stained clusters of vacuolar structures enmeshed with tubular compartments (*A*, indicated with *white rings*). Some of these stained vesicular-tubular structures are filled with stained VLPs (A, within *red* and *orange boxes*). At higher magnification, they appear as swollen tubular structures resembling ERGIC compartments (*B* and *E*, *white arrowheads*) filled with stained spherical VLPs. The spherical structures within these compartments (*C* and *D*, *red arrowheads*) resemble VLPs in size range and morphology and carry the APEX2 specific stain.

For *in situ* detection of VLPs, we coexpressed S-APEX2 with the other viral structural proteins M, N, and E. Thirty hours posttransfection, cells were fixed and processed for APEX2 TEM. Cells exhibited localized staining of various endomembrane system compartments that extended from the perinuclear region nearly to the plasma membrane (Fig. 3*A*, inset). At higher magnifications, images revealed gross perturbations of the endomembrane system with the appearance of highly stained localized vacuolar clusters enmeshed within tubular components (Fig. 3*A*, white circles). The stained areas represent localization of the S-APEX2.

VLPs were detected in some of the stained swollen vesicular-tubular structures that had the structural hallmark of the ERGIC (Fig. 3*A*, marked by red and yellow squares). Demarcated areas revealed heavily stained tubular and vesicular compartments (Fig. 3, *B* and *E*, white arrowheads) that contained spherical VLPs evident due to the presence of APEX2-tagged spike protein (Fig. 3, *C–D*, red arrowheads). The VLPs in these compartments are well within the expected size range of SARS-CoV-2 virions. Spike protein alone cannot form VLPs, indicating the proper coexpression of at least some of the other viral structural proteins.

### SARS-CoV-2 VLPs can be used to model viral entry

**Figure 4:**
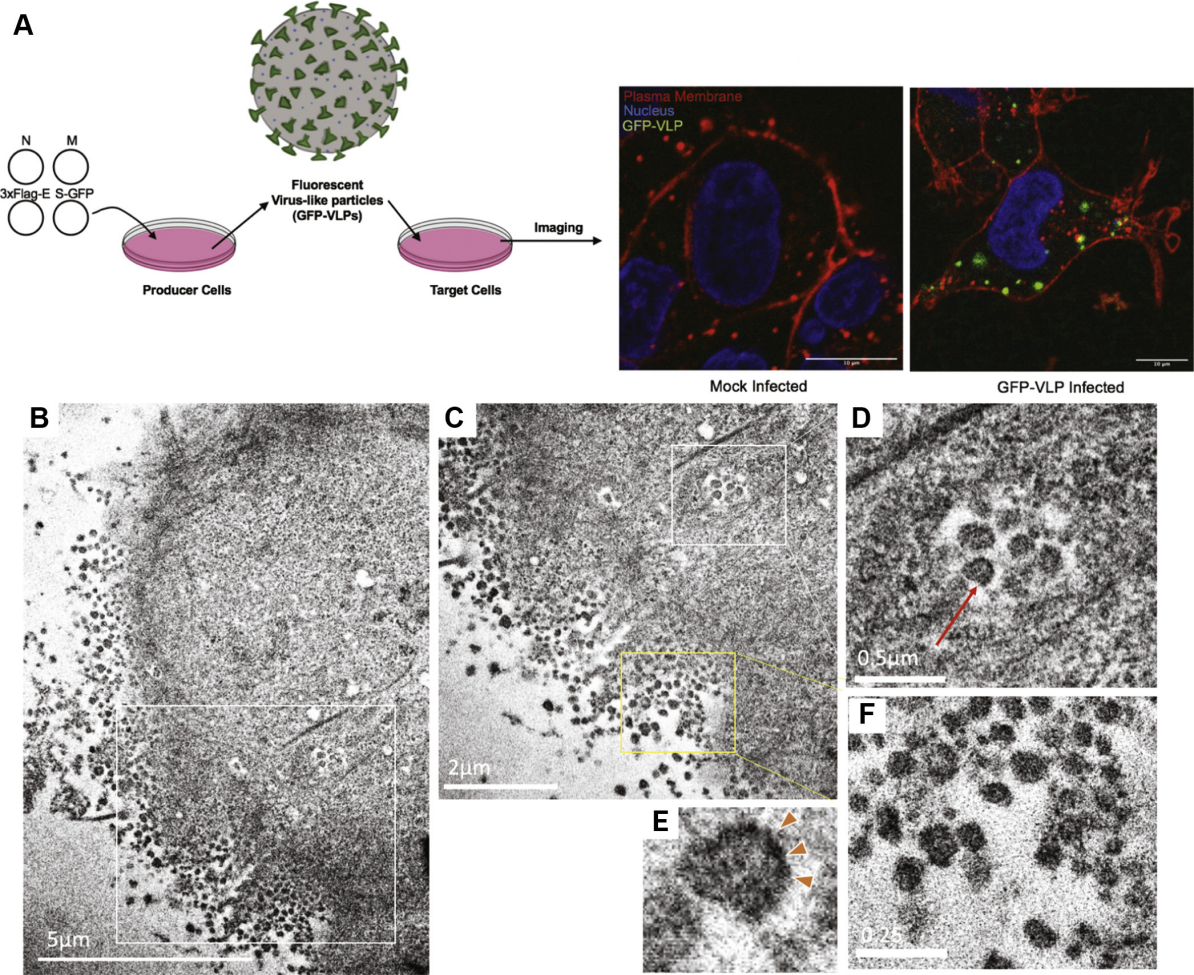
**Detection of VLP entry of target cells with confocal and electron microscopy.***A*, schematic of the GFP-VLP entry assay. GFP-VLPs were produced in HEK293 cells and used to infect target cells. After spinoculation and 2-h incubation, cells were fixed, stained with plasma membrane (WGA-Alexa647) and nuclear (Hoechst 3342) stains, and imaged on the confocal microscope. *B*, APEX2-VLPs were produced in HEK293 cells and used to infect target cells. After spinoculation and 2-h incubation, cells were fixed, processed, and imaged with TEM. APEX reaction was performed on coverslips and blocks sectioned *en face* to be able to image stained VLPs at the cell periphery (*B*, and magnified image of the area within *white box* in *A*, shown in *C* and *F*). At this stage, endosomes filled with stained VLPs are observed entering the cell (indicated with a *white box* in *C* and at higher magnification in *D*). *E*, at higher magnification, these stained structures are seen to have a darker stained periphery as expected of VLPs with stained S protein (*D*, *red arrow* and magnified image of the same in *E*). *F*, magnified image of the stained VLPs at the periphery of the cell shows a size distribution that falls within the expected range reported for SARS-CoV-1 and SARS-CoV-2 virus.

To produce fluorescently labeled VLPs, we coexpressed S-GFP with M, N, and E in HEK293 cells. S-GFP incorporation into VLPs (GFP-VLPs) collected 72-h posttransfection was confirmed with western blot analysis (Fig. S7). The S-GFP incorporated into VLPs was likely successfully processed by host furins (Fig. S7), suggesting these VLPs may be entry competent. To test the entry competency of GFP-VLPs, the GFP-VLP entry assay was performed (Fig. 4*A*, schematic). Target cells infected with GFP-VLPs had clear GFP signal present in punctate, intracellular structures while mock infected cells lacked detectable GFP signal (Fig. 4*A*). As an additional control, media collected and clarified from cells expressing S-GFP alone was collected and used to infect target cells (Fig. S8). Similar to mock infection conditions, media from S-GFP expressing cells failed to yield detectable GFP signal in infected target cells.

To model viral entry using TEM, we utilized S-APEX2 incorporated VLPs. Using the same methodology from the GFP-VLP entry assay, an APEX2-VLP entry assay was performed. Infected target cells were fixed, processed, and imaged with TEM as previously described in assembly and budding (Fig. 4, *B*–*F*). Dark staining represents the APEX2 signal and thus the localization of the APEX-VLPs. They were clearly detected as clustered in large internalized vesicles at the periphery of the cell.

### VLP entry correlates with authentic live virus entry

Coronaviruses in general are understood to utilize the host endocytic pathway to gain entry into target cells (8). Recently, SARS-CoV-2 viral particles were shown to colocalize with endocytic markers early endosome antigen 1 (EEA1) and lysosomal associated membrane protein 1 (LAMP1) after a 90-min incubation with target cells (21). Viral particles were more frequently colocalized with LAMP1+ vesicles than EEA1+ vesicles, indicating that at 90-min postinfection, most particles are already trafficked deep into the endocytic pathway.

**Figure 5:**
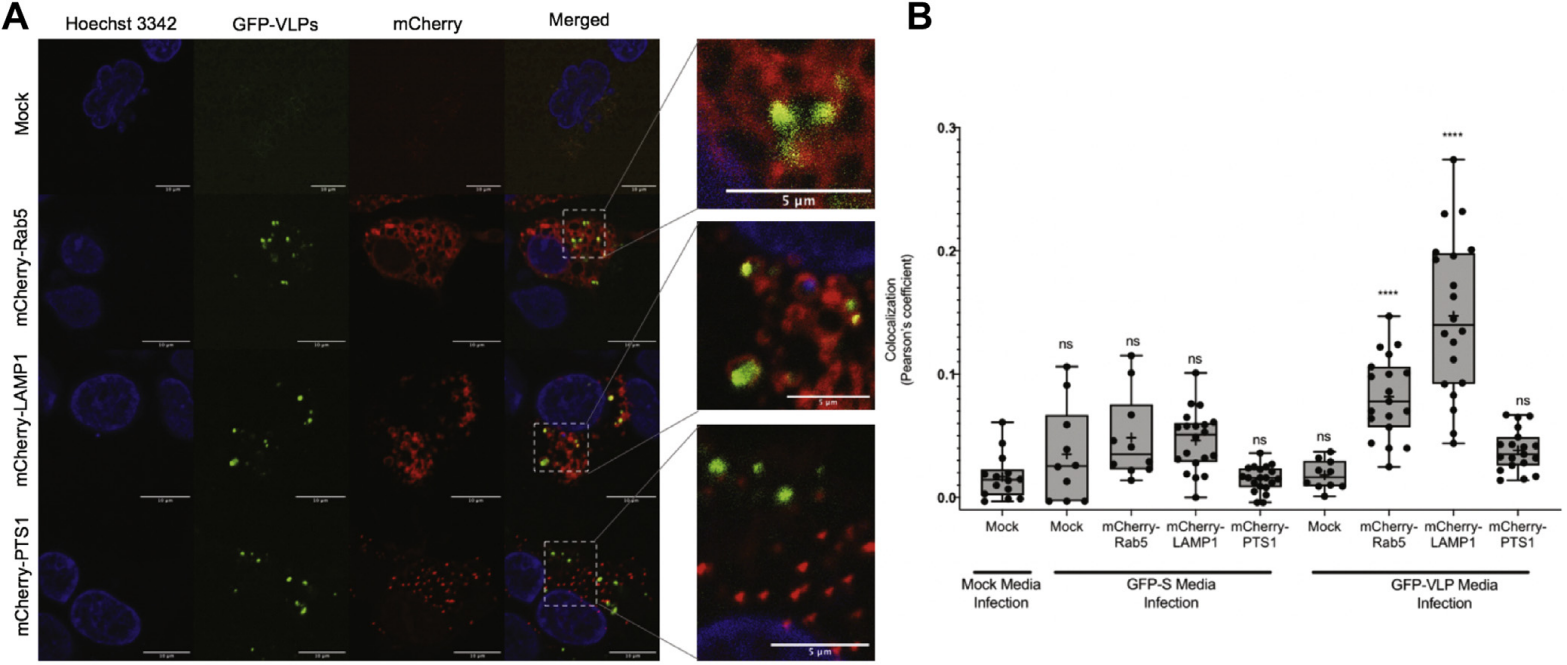
**Colocalization of GFP-VLPs with endocytic markers**. *A*, GFP-VLPs were produced and used to infect target cells coexpressing hACE2 and an mCherry-tagged marker for early endosomes (Rab5), lysosomes (LAMP1), or peroxisomes (PTS1). After infection, target cells were stained with Hoechst 3342 nuclear stain, fixed with 4% PFA, and then imaged with confocal microscopy. *B*, images were analyzed for green/red colocalization using the JACoP plug-in for ImageJ to calculate Pearson’s coefficient and graphed with Prism. Statistics were calculated using an ordinary one-way ANOVA with multiple comparisons to mock infection of mock transfected target cells. GFP-VLP infection of mCherry-Rab5 and mCherry-LAMP1 was found to have significant colocalization (∗∗∗∗) between the GFP and mCherry channels.

To evaluate the entry mechanism of our SARS-CoV-2 GFP-VLPs and compare it with that of the live virus, we performed the GFP-VLP entry assay on target cells pretransfected with endocytic pathway markers mCherry-Rab5 (early endosomes) and mCherry-LAMP1 (lysosomes) (Fig. 5*A*). Images were subsequently analyzed for green/red colocalization using the JACoP plug-in for ImageJ to calculate Pearson’s coefficient (Fig. 5*B*). Pearson’s coefficient can be used to measure colocalization between two channels with values ranging from 1 for perfectly correlated fluorescence intensities to –1 for perfectly, but inversely related fluorescence intensities. Near 0 are values that reflect two channels with intensities that are uncorrelated to each other (22).

As controls, media from mock and S-GFP expressing producer cells were used to infect mock or endocytic marker-expressing target cells, which were imaged and analyzed for green red colocalization. Colocalization analysis shows no significant colocalization between GFP and mCherry signals under any of these negative control conditions. When GFP-VLPs were used to infect mCherry-LAMP1 expressing target cells, GFP and mCherry signals colocalized with an average Pearson’s coefficient of 0.137, which was statistically significant when compared with GFP-VLP infection of mock transfected cells (0.021). When used to infect mCherry-Rab5 transfected target cells, GFP-VLPs and early endosomes had an average colocalization of 0.079, which was also statistically significant when compared with mock. When used to infect mCherry-PTS1 transfected target cells, GFP-VLPs and peroxisomes had an average colocalization of 0.036, which was not statistically significant when compared with the mock. Thus, SARS-CoV-2 VLPs localized with Rab5 positive and LAMP1 positive puncta as previously reported for authentic SARS-CoV-2. These VLP systems represent a novel approach for examining SARS-CoV-2 entry mechanisms in a BSL-2 setting.

## Discussion

As SARS-CoV-2 continues to spread, it is imperative that we continue to grow our fundamental understanding of its molecular virology. In this work, we examined the ability of viral structural proteins to produce VLPs and found that M alone was not sufficient to support VLP formation, but coexpression of M with N or S was the minimal requirement for VLP formation. E protein was found to enrich VLP production, highlighting the important role E must play in viral assembly and release. Additionally, this highlights the need for further examination of the role of E during infection. Finally, addition of S (to M + N or M + N + E) expressing cells further enriched VLP production, suggesting that M + N + E + S is the most efficient SARS-CoV-2 system for VLP production.

These findings are partially in contrast to the findings of Xu *et al* (12); however, the controversy over the minimal efficient system for SARS-CoV-2 VLP production is paralleled by the controversy over SARS-CoV-1 VLP production. One study of SARS-CoV-1 VLPs suggests that the minimal requirement for efficient VLP production was M + E (23), while another study showed that the minimal system was M + N (1). Additionally, Siu *et al* showed that the most efficient system for SARS-CoV-1 VLP production was M + N + E. As for MERS and other coronaviruses such as mouse hepatitis virus, bovine coronavirus, infectious bronchitis virus, and transmittable gastroenteritis virus, M + E was found to be the minimal system for efficient VLP production (1, 24).

While N was shown to be important in increasing VLP formation, in this work we also show that N drives the formation of filamentous filopodia in transfected cells. These findings compliment authentic live virus data, which recently detected the formation of filopodia in SARS-CoV-2 infected cells (16). It is hypothesized that these filopodia help progeny virus particles travel to and infect adjacent cells, which is supported by our SEM imaging that revealed large numbers of viral particles released at the base of filopodia. Taken together with data showing N in the insoluble fraction of HEK293 cells, this suggests that N may have lipid-binding properties; something that we plan to address in future studies.

In this work, we also present for the first time a realistic model of SARS-CoV-2 viral entry available in a BSL-2 setting: SARS-CoV-2 GFP- and APEX2-VLPs. In accordance with live virus data, GFP-VLPs colocalize with the early endosome marker, Rab5, and the late endosome marker, LAMP1. In future work, we plan to miniaturize our GFP-VLP entry assay and use it to screen for viral uptake and entry inhibitors. Not only is confocal microscopy available for evaluation of GFP-VLP entry events, we utilized APEX tagging technology to make evaluation of SARS-CoV-2 entry accessible to electron microscopy.

Traditionally, TEM has been used to demonstrate VLP-like structures in large vacuoles in cells transfected with plasmids encoding structural proteins; however, many times such identification was based solely on morphology (25). By utilizing APEX tagging, we have shown for the first time localization of S protein during VLP assembly and budding as well as the formation and export of APEX2-VLPs from the presumed ERGIC lumen.

In total, this research provides ample resources for other BSL-2 laboratories interested in joining the growing field to try and understand SARS-CoV-2 assembly, budding, and entry dynamics, biochemical and biophysical questions on the four structural proteins, and drug screening of viral assembly, budding, and/or entry inhibitors.

## Experimental procedures

### Plasmid constructs

The pcDNA3-Membrane, pcDNA3-HA-Membrane, pcDNA3-Nucleoprotein, and pCMV 3xFlag-Envelope plasmids were a kind gift from Erica Sapphire (The La Jolla Institute of Immunology, La Jolla, CA). The pCAGGS-Spike plasmid was from BEI Resources (NR-52310). The pcDNA3.1 Spike-GFP plasmid (Genescript, MC_0101089) was a generous gift to us by Raluca Ostafe (Purdue University). The pcDNA3.1-Spike-APEX2 plasmid was synthesized by Gene Universal (Newark, DE, USA). All plasmids encoding viral structural proteins were codon optimized. Sequences used of the structural protein constructs are reported in Figure S9. mCherry-Lysosomes-20 (*i.e.*, LAMP1, Addgene #55073), mCherry-Rab5a-7 (Addgene #55126), and mCherry-Peroxisomes (Addgene #54520) were gifts from Michael Davidson. pcDNA3.1-hACE2 was a gift from Hyeryun Choe (Addgene plasmid #1786) of Scripps Research, Florida (26).

### Cells and culture conditions

Human embryonic kidney (HEK293) cells (from American Type Cell Collection, Manassas, VA) were maintained in Dulbecco’s modified Eagle’s medium (DMEM) supplemented with 10% fetal bovine serum (FBS), 1% penicillin-streptomycin, and 1% MEM nonessential amino acids maintained at 37 °C and 5% CO_2_ conditions.

### Transient transfections

Transfections were performed using 2.5 M CaCl_2_ and 2X HBS [10 mM D-Glucose, 40 mM HEPES, 10 mM KCl, 270 mM NaCl, 1.5 mM Na_2_HPO_4_, pH = 7.06] as published by Abcam (https://www.abcam.com/protocols/calcium-phosphate-transfection-protocol). Briefly, DNA was mixed in ddH_2_O, CaCl_2_ was added, and the tube was mixed lightly. 2X HBS was then added to the tube for 1X final concentration, dropwise. Subsequently, the solution was mixed well and incubated at room temperature for 20 min. Transfection mixtures were added dropwise on to cells in DMEM +10% FBS and incubated for the specified time.

### Production of SARS-CoV-2 VLPs

HEK293 cells 70% confluent in 100 mm dishes were transfected with 6 μg of each SARS-CoV-2 pcDNA3-M, pcDNA3-N, pCVM-3xFlag-E, and/or pCAGGS-S. Transfection mixtures were prepared in ddH_2_O using 100 ng/μl stock DNA combined with transfection reagents (2.5 M CaCl_2_ and 2X HEPES buffered saline), incubated for 20 min at room temperature, then added dropwise to cells in DMEM +10% FBS +1% PS + 1% MEM nonessential amino acids. Cells used for the production of VLPs are referred to as “producer cells.”

### Purification of SARS-CoV-2 VLPs

Similar to methods described for the purification of SARS-CoV-1 VLPs (1), SARS-CoV-2 VLPs were purified from the media of producer cells 72-h posttransfection. Media was removed from cells and clarified with light centrifugation at 1000*g* for 10 min at room temperature. Clarified VLP-containing media was loaded on top of a 20% sucrose cushion using a glass pipette and then ultracentrifuged for 3 h at 4 °C and 100,000*g* in a Beckman Type 70 Ti rotor. VLP-containing pellets were carefully resuspended in TNE buffer (50 mM Tris-HCl, 100 mM NaCl, 0.5 mM EDTA, pH = 7.4) containing 5% sucrose. Cell lysates of producer cells were prepared using 500 μl RIPA (25 mM Tris, 150 mM NaCl, 1% Tween 20, 0.5% sodium deoxycholate, 0.1% SDS) buffer containing 1X Halts protease inhibitor and 0.5% N-Lauroylsarcosine for 1 h on ice, vortexing every 15 min, followed by a 10-s sonication and finally ultracentrifugation at 25,000*g* for 20 min at 4 °C; the soluble fraction was collected and the pellet was resuspended in 500 μl RIPA buffer containing 1X Halts protease inhibitor and 0.5% N-Lauroylsarcosine by sonicating for 10 s.

### Western blot analysis of VLPs and cell lysates

Total protein content of producer cell lysates was used to normalize loading conditions and was quantified using the Pierce bicinchoninic acid assay (BCA). VLP loading was calculated as a constant ratio to normalized cell lysates. Samples were prepared in 1X E running buffer (5 M Urea, 1 M DTT, 100 mM NaCO_2_, 25 mM Tris, 0.5% CHAPS, pH = 11) with 1X reducing Laemmli SDS Sample buffer (Alfa Aesar), resolved in a 10% SDS-PAGE polyacrylamide gel, and subsequently transferred on to a supported 0.45 μm nitrocellulose membrane (BioRad), which was used for immunoblotting. Membranes were cut and probed for M with rabbit anti-SARS-1 M (Rockland), N with rabbit anti-SARS-2 N (Genetex), 3xFlag-E with mouse anti-Flag IgG HRP (Abcam), and S with mouse anti-SARS1/2-Δ10S [1A9] (Genetex); goat anti-rabbit IgG HRP (Abcam) and sheep anti-mouse IgG HRP (Abcam) secondary antibodies were used as appropriate. GAPDH was used as a cell lysate loading control by probing membranes with mouse anti-GAPDH monoclonal IgG (Thermofisher Scientific) followed by sheep antimouse IgG HRP. All antibodies were diluted in 5% milk and membranes were washed with 1X TBST. Chemiluminescent signal was visualized using Clarity Western ECL Substrate (BioRad) or Clarity Max Western ECL Substrate (BioRad) and imaged using an Amersham Imager 600 (GE Healthcare Life Sciences).

### Immunofluorescence

HEK293 cells of 70% confluency grown in 8-well glass bottom plates were transfected with pcDNA3-N using either lipofectamine LTX or 2000 reagent (Thermofisher scientific) according to the manufacturer’s instructions. At 24-h posttransfection, the cells were fixed by 4% paraformaldehyde (PFA), permeabilized by 0.1–0.2% Triton X-100 in PBS, blocked by 2.5–3% FBS, and 1% BSA in PBS and then incubated with the appropriate primary antibody overnight (Rabbit anti-SARS-CoV-2-N antibody, GeneTex #GTX135357, 1:500 or Rabbit anti-SARS-1-M, Rockland, 1:1000; mouse anti-GORAPS2 (Golgi marker), Sigma Aldrich, 1:500). Following overnight incubation, cells were washed and then incubated with the appropriate secondary antibody (anti-rabbit IgG-Atto 594, Millipore Sigma #77671-1Ml-F, 1:1000; antimouse IgG-Atto488, Millipore, 1;10,000) for 45 min to 1 h at room temperature. Following secondary incubation, cells were washed, stained with Hoechst 3342 nuclear stain, and then imaged with confocal microscopy. Images were analyzed using ImageJ. For images of N protein alone, some cell body areas were saturated to achieve filopodia visibility.

### Negative stain transmission electron microscopy

To prepare the grids for negative stain EM, 4 μl of purified VLPs was added to a glow discharged 400-mesh copper grid covered with carbon-coated collodion film (EMS, Hatfield, PA). Grids were washed in one drop of water, stained in three drops of Phosphotungstic acid (1.0% w/v) (EMS, Hatfield, PA), and air dried. Samples were visualized on a Tecnai G2 T20 electron microscope (FEI, Hillsboro, OR) at an acceleration voltage of 200 kV. Images were taken at a magnification of 43,000× at a defocus value of –1.4 μm and recorded on a Gatan US1000 2K x 2k CCD camera (Gatan, Pleasanton, CA). Images were converted to mixed raster content format, resulting in final images with a pixel size of 4.23 Å/pixel at the specimen level.

### Scanning electron microscopy

Silica coverslips were placed on the bottom of 12-well plates before being seeded with HEK293 cells to 30% confluency for transfection 24-h after seeding. Calcium chloride transfection was conducted with structural protein cDNA vectors and cells were incubated in DMEM + 10% FBS for 72 h. At 72 h posttransfection, cells were fixed with primary fixative, 2.4% glutaraldehyde, 0.1 M cacodylate fixative buffer and sealed in parafilm at 4 °C. Samples were then processed by the Purdue Electron Microscopy Facility, washing coverslips with 0.1 M cacodylate buffer before the addition of secondary fixative, 4% osmium tetroxide, 0.1 M cacodylate buffer and incubated for 30 min. Samples were then dehydrated gradually with increasing percentage of ethanol and then dried in the critical point dryer (Tousimis AutoSAMDRI-931, CPD) available at the facility. After drying, coverslips were mounted onto aluminum pin stub mounts with double-sided conductive tape, conductive liquid silver paint, and sputter coated for 60 s. Upon completion of the sample preparation, samples were visualized and imaged on the FEI Nova NanoSEM at the Purdue Life Science Electron Microscopy Facility.

### GFP-VLP entry assay

Following the protocol outlined in *Production of SARS-CoV-2 VLPs*, SARS-CoV-2 pcDNA3-M, pcDNA3-N, pCVM-3xFlag-E, and pcDNA3.1-S-GFP were coexpressed in HEK293 cells. Seventy-two hours posttransfection, media was collected from producer cells and clarified with light centrifugation at 1000*g* for 10 min at room temperature. Five milliliters of clarified media was added per well to 70% confluent target HEK293 cells plated in a black, glass-bottom 6-well plate (Cellvis, Mountain View, CA). Target cells were then spinoculated using an M-20 microplate swinging bucket rotor at 2000 rpm, 4 °C, for 1 h. After spinoculation, target cells were placed at 37 °C for 2 h. For imaging-based experiments, following this incubation, infected target cells were stained with Hoechst 3342 nuclear stain and wheat germ agglutinin (WGA)-Alexa 647 plasma membrane stain, fixed with 4% paraformaldehyde for 7 min at room temperature, and then imaged with confocal microscopy.

### APEX2-VLP entry assay

Following the protocol outlined in *Production of SARS-CoV-2 VLPs*, SARS-CoV-2 pcDNA3-M, pcDNA3-N, pCVM-3xFlag-E, and pcDNA3.1-S-APEX2 were coexpressed in HEK293 cells. Seventy-two hours posttransfection, media was collected from producer cells and clarified with light centrifugation at 1000*g* for 10 min at room temperature. Five milliliters of clarified media was added per well to 70% confluent target HEK293 cells transiently expressing hACE2 plated on 22 mm glass coverslips in a 6-well plate. Target cells were then spinoculated using an M-20 microplate swinging bucket rotor at 2000 rpm, 4 °C, for 1 h. After spinoculation, target cells were placed at 37 °C for 2 h. Postincubation, cells were fixed with 2.5% glutaraldehyde in 0.1% sodium cacodylate buffer (pH 7.4) and were kept on ice for 30 min. Cells were kept between 0 and 4 °C for all subsequent steps until resin infiltration. Cells were washed five times, 3 min each, with chilled cacodylate buffer and incubated in 1 ml of freshly prepared 0.5 mg/ml DAB (Sigma-Aldrich) for 2 min. DAB combined with 10 mM of hydrogen peroxide was then added to the cell and incubated for 15 min or until a dark brown color developed. DAB was removed and the cells were washed three times, 5 min each, followed by staining with 1% Osmium tetroxide for 10 min on ice. Cells were washed two times, 5 min each, in chilled cacodylate buffer and twice with water. Cells were then dehydrated in a graded ethanol series (50%, 70%, 90%, 95%, 100%, 100%, 100%), for 10 min each and then infiltrated with an increasing concentration of Durcupan ACM resin (Sigma-Aldrich) in ethanol (30%, 60%, 90% 2 h each and then overnight in 100% and 2 h twice in 100% + component C) with gentle rocking. The coverslips with cells on them were then picked up with tweezers and planted face down on BEEM capsules (Electron Microscopy Sciences) prefilled with 100% + C and baked in the oven at 60 °C for 36 h. Coverslips were separated from the BEEM capsules by dipping them in liquid nitrogen. Blocks were then extracted from the BEEM capsules, loaded onto the ultramicrotome. Ninety nanometer sections were obtained *en face* from the single layer of cells using a diamond knife (DiATOME) and imaged on a T12 (FEI) TEM.

### TEM-based subcellular localization of VLP assembly using APEX TEM

Subcellular localization of tagged VLPs was visualized using the APEX method as described in Martell *et al.* (20). Following the protocol outlined in *Production of SARS-CoV-2 VLPs*, SARS-CoV-2 pcDNA3-M, pcDNA3-N, pCVM-3xFlag-E, and pcDNA3.1-S-APEX2 were coexpressed in HEK293 cells. At 30-h posttransfection, cells were fixed with 2.5% glutaraldehyde and 2% sucrose in 0.1% sodium cacodylate buffer (pH 7.4) on ice for 30 min. The HEK293 monolayer on the coverslips was then processed exactly as described before in *APEX2-VLP entry assay* and sectioned *en face*, screened and imaged on the T12 (FEI) electron microscope operating at 80 kV.

### GFP-VLP colocalization with endocytic markers

Following the protocol outlined in *GFP-VLP Entry Assay*, SARS-CoV-2 GFP-VLPs were prepared in HEK293 cells. Seventy-two hours posttransfection, media was collected from producer cells and clarified with light centrifugation at 1000*g* for 10 min at room temperature. Five milliliters of clarified media was added per well to 70% confluent target HEK293 cells plated in black, glass-bottom 6-well plates (Cellvis, Mountain View, CA), which had been transfected approximately 16-h previously with 1 μg hACE2 and 1 μg mCherry-LAMP1, 1 μg hACE2 and 1 μg mCherry-Rab5, 1 μg hACE2 and 1 μg mCherry-PTS1, or mock. Target cells were then spinoculated using an M-20 microplate swinging bucket rotor at 2000 rpm, 4 °C, for 1 h. After spinoculation, target cells were placed at 37 °C for 2 h. Following this incubation, cells were stained with Hoechst 3342 nuclear stain and wheat germ agglutinin (WGA)-Alexa 647 plasma membrane stain, fixed with 4% paraformaldehyde for 7 min at room temperature, and then imaged with confocal microscopy.

### Fluorescence microscopy

All confocal imaging was performed using the Purdue College of Pharmacy Live Cell Imaging Facility Nikon Eclipse Ti A1 instrument using NIS-elements AR software to capture 1024 × 1024 pixel resolution images at 1/4 frame/second on 60× oil objectives detecting the fluorophores with channels in series.

## Data availability

The majority of data are contained within the article. For data not included within the article, data can be shared by contacting the corresponding author.

## Conflict of interest

The authors declare that they have no conflicts of interest with the contents of this article.
