# Supplementary material for: SARS-CoV-2 viral budding and entry can be modeled using BSL-2 level virus-like particles
Source: J Biol Chem. 2020 Nov 27;296:100103. doi: 10.1074/jbc.RA120.016148 (PMC7832013; doi:10.1074/jbc.RA120.016148)
Supplement: Supplementary file 1 — Figures S1 to S9 [file mmc1.pdf]

Supporting Information for

SARS-CoV-2 viral budding and entry can be modeled using BSL-2 level virus-like particles

Caroline B. Plescia, Emily A. David, Dhabaleswar Patra, Ranjan Sengupta, Souad Amiar, Yuan Su, and Robert V. Stahelin*

*Corresponding Author: Robert V. Stahelin
E-mail: rstaheli@purdue.edu

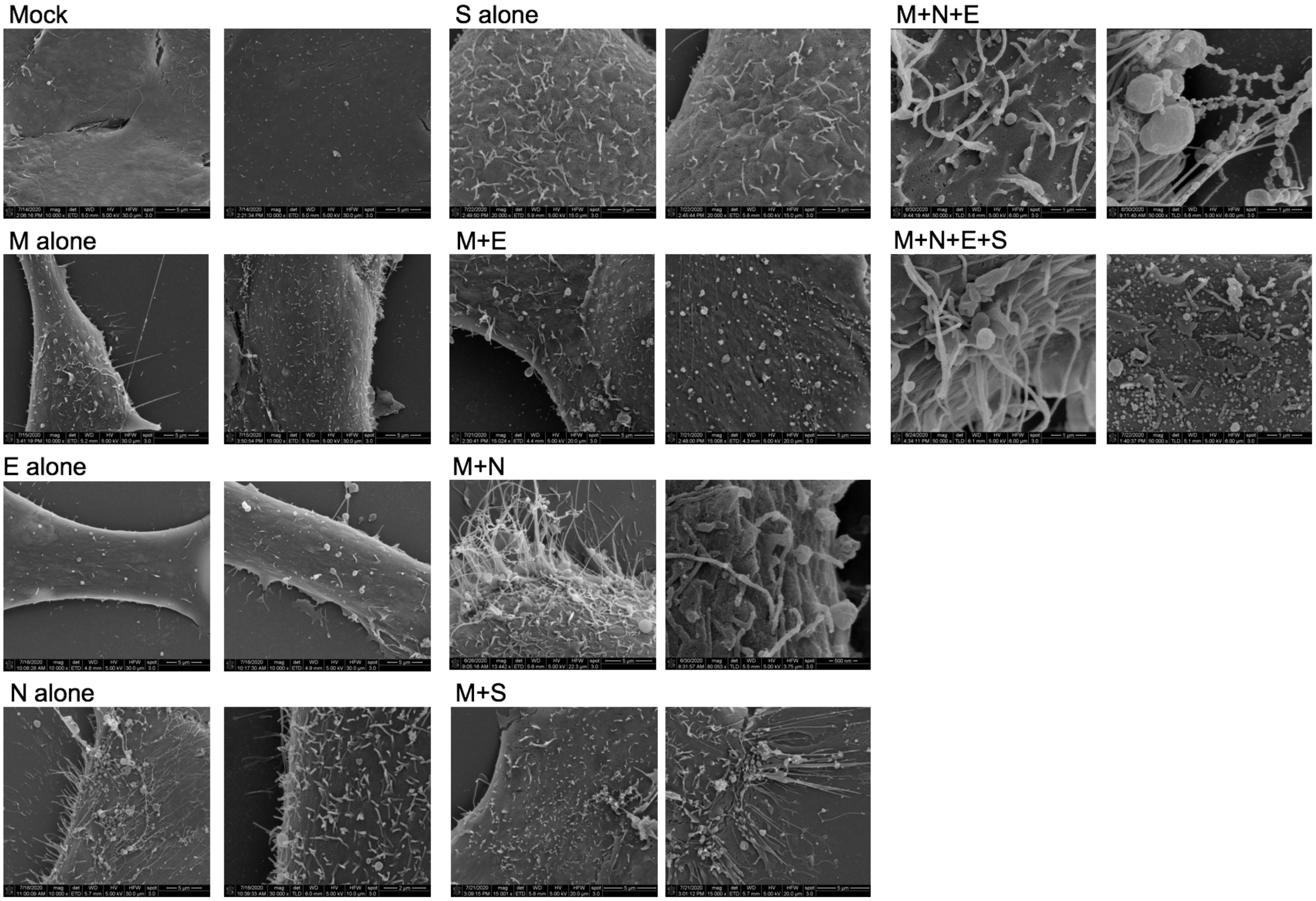

Figure S1. Scanning electron microscopy of SARS-CoV-2 viral structural protein expressing cells. HEK293 cells were seeded onto coverslips and transfected individually and in combination with M, N, E, and/or S. Cells were fixed with glutaraldehyde 72-hours post-transfection and kept at 4°C until fixed with osmium tetroxide. Samples were then gradually dehydrated with ethanol and completely dehydrated with a critical point dryer. Once dehydrated, samples were mounted onto aluminum pins with double-sided carbon tape, charged with silver paint, and sputter coated prior to imaging.

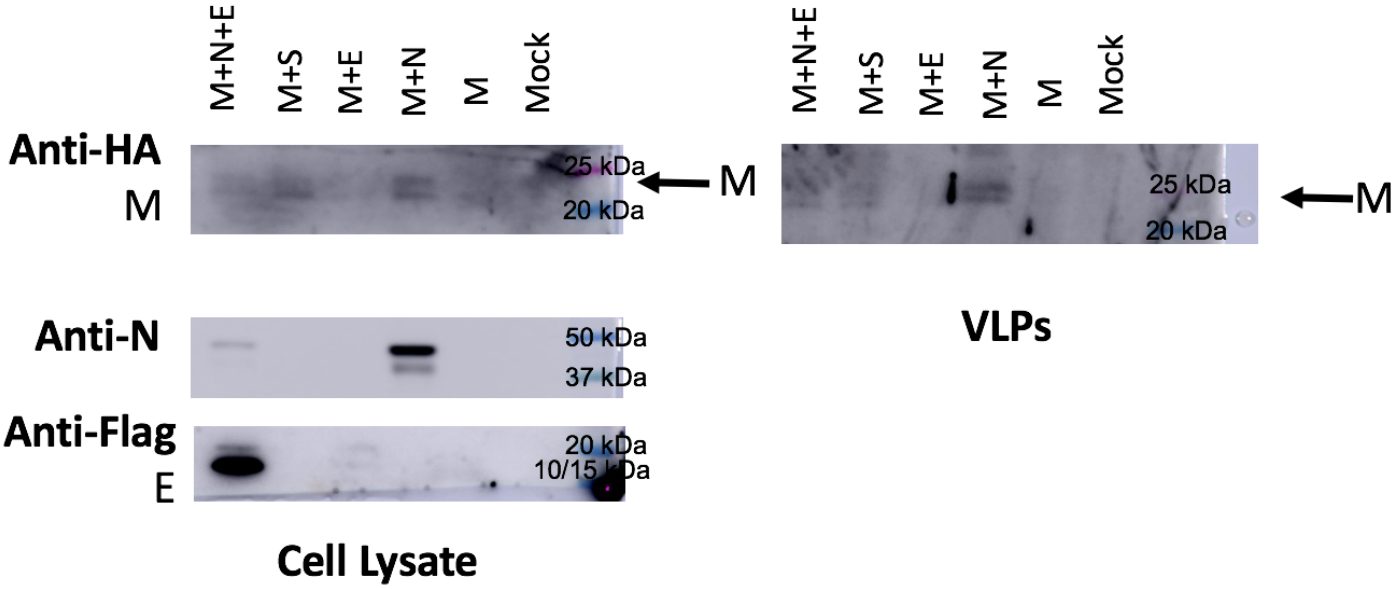

Figure S2. Western blot analysis of the cell lysate and VLP fractions of different combinations of M, N, E, and/or S. Total protein content of cell lysates were used to normalize loading conditions and was quantified using the Pierce bicinchoninic acid assay. VLP loading was calculated as a constant ratio to normalized cell lysates.

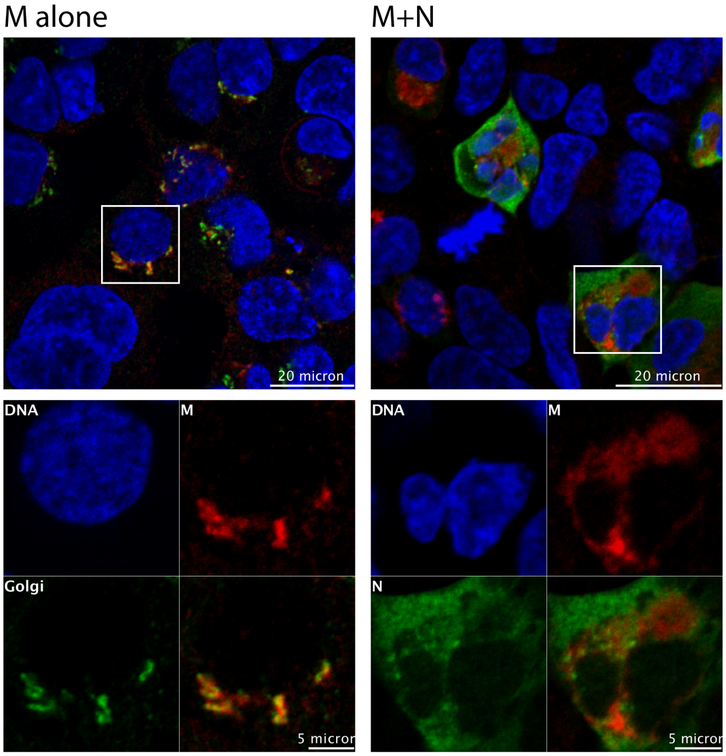

Figure S3. Cellular redistribution and accumulation of SARS-CoV-2 M protein in the presence of N protein. Immunofluorescence assays of HEK293 cells expressing M alone or M+N for 24 hours prior to fixation, indicated that M protein expressed alone accumulated at the Golgi apparatus (as indicated in the zoomed left inset). However, when co-expressed with N protein, M protein exhibited more diffuse and perinuclear distribution (as indicated in zoomed right inset).

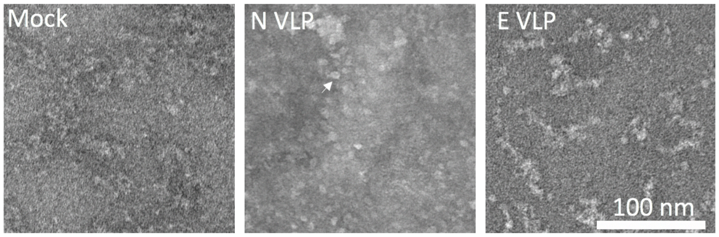

Figure S4. TEM analysis of VLP fractions. VLP collections were performed on mock, N, or E-transfected cells. Each VLP fraction was added to a glow discharged 400-mesh copper grid covered with carbon-coated collodion film. Grids were washed in one drop of water, stained in three drops of phosphotungstic acid (1.0% w/v), air dried, and imaged. Arrow in N VLP fraction represents large protein oligomers.

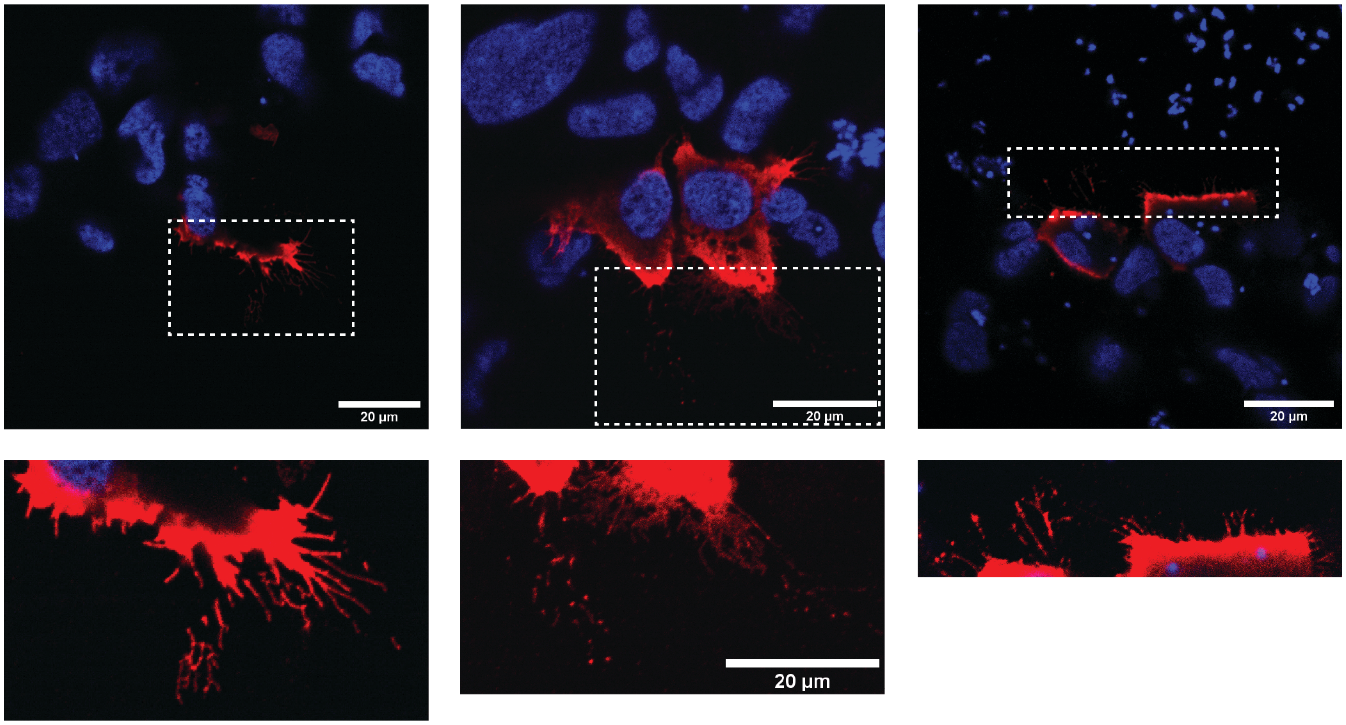

Figure S5. Immunofluorescence of SARS-COV-2 N in HEK293 cells. HEK293 cells expressing SARS-COV-2 N protein at 24-hours post-transfection were fixed, stained with nuclear stain (blue, Hoechst 3342) and subject to immunofluorescence assay with anti-SARS-COV-2 N antibody (1:500) followed by anti-rabbit IgG-Atto 594 (1:1000). SARS-COV-2 N protein localized in the filopodia along the cell surface.

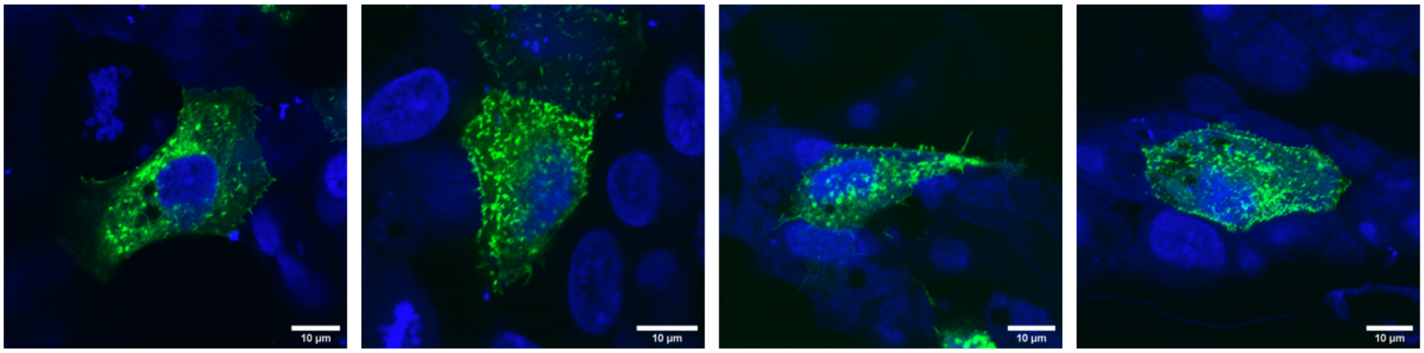

Figure S6: Expression of S-GFP in HEK293 cells. Fluorescence confocal images of SARS-CoV-2 S protein in HEK293 cells. HEK293 cells transiently expressing SARS-CoV-2 S protein at 24 hours post transfection were fixed, stained with nuclear stain (blue, Hoechst 3342) and imaged with confocal microscopy.

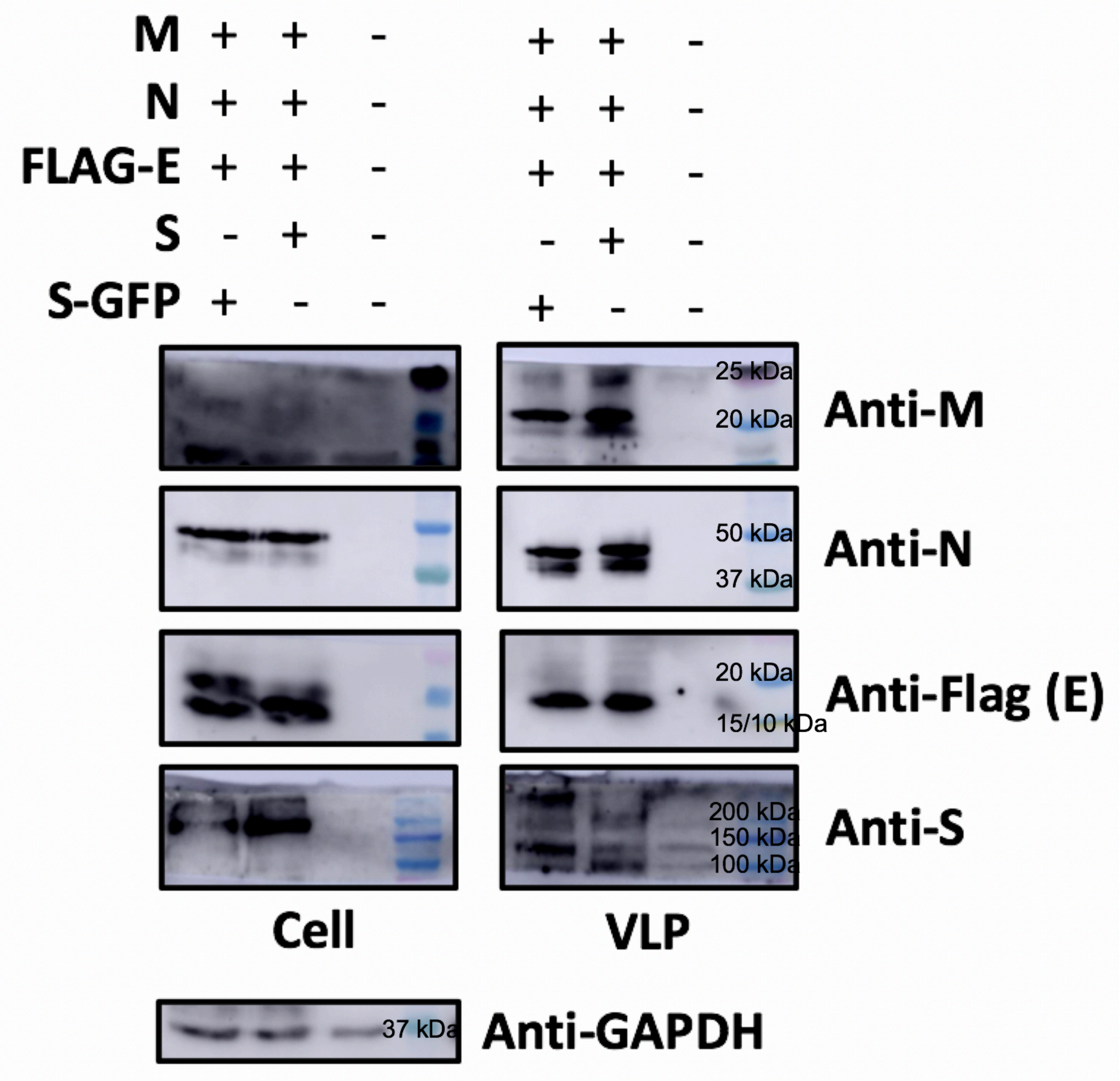

Figure S7. Western blot analysis of the cell lysate and VLP fractions of VLP and GFP-producing combinations of M, N, E, and S or S-GFP. Total protein content of cell lysates were used to normalize loading conditions and was quantified using the Pierce bicinchoninic acid assay. VLP loading was calculated as a constant ratio to normalized cell lysates.

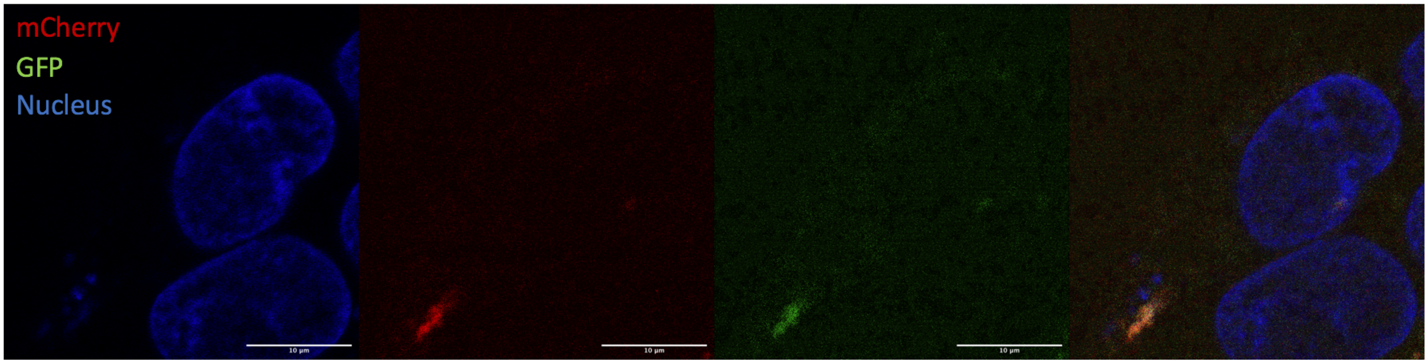

Figure S8. Infection of target HEK293 cells with media from S-GFP transfected cells. Media was removed from HEK293 cells expressing S-GFP 72-hours post-transfection and was used to infect target HEK293 cells. Target cells were spinoculated, incubated two hours, stained with nuclear stain (Hoechst 3342), and imaged with confocal microscopy.

**Plasmid**	**Construct**	**Tag**	**Construct Sequence / Reference**
pcDNA3-M	M	No Tag	ATGGCCGACAGCAACGGCACCATCACCGTGGAAGAGCTGAAGAAACTGCTGGAACAGTGGAACC<br>TGGTCATCGGCTTTCTGTTCCTGACCTGGATCTGCCTGCTGCAGTTCGCTTATGCCAACCGGAACC<br>GGTTCCTGTACATCATCAAGCTGATCTTCCTGTGGCTGCTGTGGCCTGTGACCCTGGCTTGTTTCGT<br>GCTGGCCGCCGTTTACCGCATCAACTGGATCACAGGCGGAATCGCTATCGCAATGGCCTGCCTGG<br>TGGGCCTGATGTGGCTGTCCTACTTCATCGCCAGCTTCCGGCTGTTTGCCAGAACCAGGAGCATGT<br>GGTCCTTCAACCCTGAGACAAACATCCTGCTGAACGTGCCCCTCCACGGCACAATCCTGACAAGAC<br>CACTGCTGGAGAGCGAGCTGGTGATTGGCGCCGTGATCCTTAGAGGCCACCTGAGAATCGCCGG<br>CCACCATCTGGGCAGATGCGATATCAAGGACCTGCCTAAGGAAATCACCGTGGCCACATCCAGAA<br>CCCTGAGCTACTACAAACTGGGAGCCTCTCAAAGAGTGGCCGGCGATAGCGGCTTCGCCGCTTAC<br>AGCAGATACCGGATTGGAAATTACAAGCTGAATACCGACCACTCTAGCTCTAGCGACAACATCGCC<br>CTGCTGGTGCAGTGA
pcDNA3-HA-M	HA-M	HA	TACCCATACGATGTTCCAGATTACGCTGGTACCGGCGGAAGCGCCGACAGCAACGGCACCATCAC<br>CGTGGAAGAGCTGAAGAAACTGCTGGAACAGTGGAACCTGGTCATCGGCTTTCTGTTCCTGACCT<br>GGATCTGCCTGCTGCAGTTCGCTTATGCCAACCGGAACCGGTTCCTGTACATCATCAAGCTGATCT<br>TCCTGTGGCTGCTGTGGCCTGTGACCCTGGCTTGTTTCGTGCTGGCCGCCGTTTACCGCATCAACT<br>GGATCACAGGCGGAATCGCTATCGCAATGGCCTGCCTGGTGGGCCTGATGTGGCTGTCCTACTTC<br>ATCGCCAGCTTCCGGCTGTTTGCCAGAACCAGGAGCATGTGGTCCTTCAACCCTGAGACAAACATC<br>CTGCTGAACGTGCCCCTCCACGGCACAATCCTGACAAGACCACTGCTGGAGAGCGAGCTGGTGAT<br>TGGCGCCGTGATCCTTAGAGGCCACCTGAGAATCGCCGGCCACCATCTGGGCAGATGCGATATCA<br>AGGACCTGCCTAAGGAAATCACCGTGGCCACATCCAGAACCCTGAGCTACTACAAACTGGGAGCC<br>TCTCAAAGAGTGGCCGGCGATAGCGGCTTCGCCGCTTACAGCAGATACCGGATTGGAAATTACAA<br>GCTGAATACCGACCACTCTAGCTCTAGCGACAACATCGCCCTGCTGGTGCAGTGA
pcDNA3-N	N	No Tag	ATGTCTGACAACGGCCCTCAGAACCAGCGGAATGCCCCAAGAATCACCTTCGGCGGCCCCTCCGA<br>TTCTACAGGCTCCAACCAGAATGGAGAGAGGTCCGGAGCACGCTCTAAGCAGCGGAGACCACAG<br>GGCCTGCCCAACAATACCGCCAGCTGGTTCACCGCCCTGACACAGCACGGCAAGGAGGACCTGA<br>AGTTTCCCAGGGGCCAGGGCGTGCCTATCAACACCAATAGCTCCCCTGACGATCAGATCGGCTAC<br>TATAGGAGGGCAACAAGGAGAATCCGGGGAGGCGACGGCAAGATGAAGGATCTGTCCCCCAGA<br>TGGTACTTCTACTATCTGGGCACCGGACCTGAGGCAGGACTGCCATATGGCGCCAATAAGGACGG<br>AATCATCTGGGTGGCAACCGAGGGCGCCCTGAACACACCAAAGGATCACATCGGCACACGCAAT<br>CCCGCCAACAATGCAGCAATCGTGCTCCAGCTGCCACAGGGCACCACACTGCCCAAGGGCTTTTA<br>CGCAGAGGGCAGCAGGGGCGGCTCCCAGGCCTCTAGCCGCTCCTCTAGCCGGTCCAGAAACTCC<br>TCTCGGAATTCTACCCCAGGCAGCTCCCGGGGCACAAGCCCTGCAAGAATGGCAGGAAACGGAG<br>GCGACGCCGCCCTGGCCCTGCTGCTGCTGGATAGACTGAATCAGCTGGAGTCTAAGATGAGCGG<br>CAAGGGACAGCAGCAGCAGGGACAGACCGTGACAAAGAAGTCTGCCGCCGAGGCCAGCAAGAA<br>GCCAAGGCAGAAGCGCACCGCCACAAAGGCCTACAACGTGACCCAGGCCTTCGGCAGGCGCGG<br>ACCAGAGCAGACACAGGGCAATTTTGGCGACCAGGAGCTGATCAGGCAGGGCACCGATTATAAG<br>CACTGGCCTCAGATCGCACAGTTCGCACCAAGCGCCTCCGCCTTCTTTGGCATGAGCAGGATCGG<br>AATGGAGGTGACCCCATCCGGCACATGGCTGACCTACACAGGCGCCATCAAGCTGGACGATAAG<br>GACCCTAACTTCAAGGATCAGGTCATCCTGCTGAACAAGCACATCGATGCCTATAAGACCTTTCCC<br>CCTACAGAGCCCAAGAAGGACAAGAAGAAGAAGGCCGATGAGACCCAGGCCCTGCCTCAGAGA<br>CAGAAGAAGCAGCAGACCGTGACACTGCTGCCAGCCGCCGACCTGGACGATTTTCCAAGCAGCT<br>CCAGCAGTCTATGTCTAGCGCCGATAGCACCCAGTGA
pCMV-3xFlag-E	3xFlag-E	3xFlag	ATGGATTACAAGGATGACGACGATAAGGACTATAAGGACGATGATGACAAGGACTACAAAGATGA<br>TGACGATAAAGCCCGGGCGGGATCCCCCGGGCTGCAGGAATTCGATATCAAGCTTTACTCTTTTGT<br>GAGCGAGGAGACCGGCACACTGATCGTGAACTCCGTGCTGCTGTTCCTGGCCTTTGTGGTGTTCC<br>TGCTGGTGACCCTGGCAATCCTGACAGCCCTGAGGCTGTGCGCCTATTGCTGTAACATCGTGAAC<br>GTGAGCCTGGTGAAGCCCTCTTTCTACGTGTATAGCCGGGTGAAGAACCTGAATAGCTCCAGAGT<br>GCCTGACCTGCTGGTGTGA
pCAGGS-S	S	No Tag	BEI Resources NR-52310
pcDNA3.1-S-GFP	S-GFP	GFP	Genescript MC_0101089
pcDNA3.1-S-APEX2	S-APEX2	APEX2	S-GFP construct with GFP replaced with APEX2

Figure S9. Table of construct sequences used for mammalian expression of SARS-CoV-2 structural proteins.
